# Health status, lifestyle habits, and perceived social support in long-term cancer survivors: a cross-sectional study

**DOI:** 10.1186/s13104-020-05218-8

**Published:** 2020-08-08

**Authors:** Beatriz León-Salas, Edurne Zabaleta-del-Olmo, Joan Llobera, Bonaventura Bolíbar-Ribas, Tomás López-Jiménez, Marc Casajuana-Closas, Magdalena Esteva

**Affiliations:** 1 Canarian Foundation in Health Care Research (FUNCANIS), Camino Candelaria, 44. CS San Isidro-El Chorrillo, 38109 El Rosario, Santa Cruz de Tenerife Spain; 2 Preventive Activities and Health Promotion Research Network (REDIAPP), Barcelona, Spain; 3 Health Services Research in Chronic Diseases Network (REDISSEC), Barakaldo, Spain; 4 University Institute for Research in Primary Care Jordi Gol I Gurina (IDIAPJGol), Gran Via de Les Corts Catalanes, 587, 08007 Barcelona, Spain; 5 Barcelona Health District, Catalonial Health Institute, Barcelona, Spain; 6 Faculty of Nursing, University of Girona, Girona, Spain; 7 Autonomous University of Barcelona, Bellaterra, Spain; 8 Unit of Research Majorca Department of Primary Care, Balearic Health Service, Escola Graduada 3, 07002 Palma, Majorca Spain; 9 Balearic Islands Health Research Institute (IdISBa), University Hospital of Son Espases, Carretera de Valldemossa, 79, 07120 Palma, Majorca Spain

**Keywords:** Primary care, Cancer, Survivorship, Lifestyle, Comorbidity, Quality of life, Health promotion

## Abstract

**Objective:**

To compare the presence of comorbidities and self-perceived health and social support between long-term cancer survivors and people without a history of cancer from a clinical trial examining the effects of a multiple risk behavior intervention.

**Results:**

Of the 4259 people studied, 190 (4.46%) were cancer survivors. They had a mean ± SD age of 62.8 ± 7 years vs. 58.7 ± 8 years (P < 0.01) for non-cancer people and were more likely to be on long-term sick leave (11.9 vs. 3.5%, P < 0.001). No differences were observed for smoking, adherence to the Mediterranean diet, physical activity, obesity, or social support. Cancer survivors were more likely to have worse self-perceived health (OR 1.82; 95% CI 1.02–2.75), more comorbidities (OR 1.68; 95% CI 1.18–2.39), COPD (OR 2.17; 95% CI 1.25–3.78), and depression (OR 1.65; 95% CI 1.06–2.57). Older age and worse self-perceived health were independent predictors of survivorship in the adjusted analysis.

## Introduction

More and more people are surviving cancer thanks to advances in early detection and diagnostic techniques and treatment. Survival rates are increasing worldwide, even for the most aggressive forms of cancer [1]. In Spain, over 50% of adults are still alive 5 years after diagnosis [2] and the estimated number of prevalent cases at 5 years exceeds 500,000 [3]. These trends call for greater attention to be paid to long-term cancer survivorship.

As explained by Grunfel and Earle [3], “the period after completing primary and adjuvant cancer treatment until recurrence or death is now recognized as a unique phase in the cancer control continuum. Survivorship is a time of transition: Issues related to diagnosis and treatment diminishes in importance, and concerns related to long-term follow-up care, management of late effects, rehabilitation, and health promotion dominate.”

Cancer survivors may develop other conditions as a result of their cancer treatment. Some of these are transient, but others can become chronic and significantly affect quality of life [4]. Cancer survivors may also be at increased risk of a second cancer if the risk factors associated with the original cancer persist. There is increasing evidence that interventions aimed at promoting healthy eating, regular exercise, and maintenance of a healthy weight can offset some of the adverse effects of cancer and cancer treatment [5–10]. Alongside smoking cessation, these behaviors reduce not only the risk of cancer recurrence but also the risk of other health conditions, such as cardiovascular disease, diabetes, and other cancers [11–13]. Cancer also has social impacts in the form of job loss or change and difficulties with social and personal relationships [14]. Although the health and quality of life of cancer survivors are important public health issues, knowledge in this area is still lacking, particularly in Spain [15].

The aim of this study was to compare the presence of comorbid conditions and self-perceptions of health and social support between long-term cancer survivors and people without a history of cancer from a clinical trial examining the effects of a multiple risk behavior intervention.

## Main text

### Methods

We performed a cross-sectional multicenter study nested within phases II and III of the ERIA trial, a parallel cluster randomized clinical trial designed to analyze the effects of a complex individual and group multiple risk intervention on the uptake of healthy behaviors (smoking cessation, adherence to the Mediterranean diet, and sufficient levels of physical activity). The trial was conducted in accordance with the Medical Research Council guidance on complex interventions [16]. The definitive (phase III) trial was launched after the exploratory phase II trial. It was conducted between 2014–2015 and 2016–2018 by the Primary Care Prevention and Health Promotion Network (RedIAPP) in 38 primary care centers in 11 Spanish provinces. The trial protocol has been published [17].

The participants were recruited by their general practitioners (GPs). Inclusion criteria were: (1) be aged between 45 and 75 years, (2) be registered with a GP participating in the study and have had arranged an appointment with their GP or nurse, and (3) have two of the following risk behaviors: smoking, low adherence to the Mediterranean diet, and insufficient physical activity. Cardiovascular risk and depression had been used as inclusion criteria in the exploratory phase II trial, but were eliminated in the phase III trial due to feasibility issues. Exclusion criteria were cognitive impairment, dependency in activities of daily living, severe mental disease, long-term home care and active or palliative cancer treatment. The flow of participants through the study is shown in Additional file 1: Figure S1.

Based on the assumption that 50% of cancer survivors and 40% of non-cancer patients would rate their health as fair or poor, we estimated that the study would be powered at 78% with a confidence level of 95%.

For this sub-study, the variables analyzed were those measured at the baseline interview (i.e., before any intervention). The dependent variable was presence or absence of a cancer diagnosis. The subjects were thus divided into cancer survivors (patients diagnosed with cancer who met the criteria for survivorship) and patients without a history of cancer). Cancer survivorship was defined as the period from completion of primary or adjuvant cancer treatment to recurrence, death, or cure. ClinicalTrials.gov, NCT03136211.

Other baseline variables were (1) sociodemographic variables, namely age, sex, level of education, civil status, and employment status, classified as working/not working (homemaker, retiree, student) unemployed and long-term sick leave; (2) perceived social support measured using the Duke-UNC questionnaire [18, 19], (< 32 points indicating low support); (3) self-perceived health status; (4) comorbidity measured using the non-cancer Charlson Comorbidity Index (CCI) [20]; (5) lifestyle habits, namely smoking (yes/no); adherence to the Mediterranean diet measured using the 14-item PREDIMED scale [21], where 0–8 points indicates low adherence) [5]; and physical activity, assessed using the Brief Physical Activity Assessment tool for primary care [22, 23], where patients are classified as *sufficiently active* if they perform three or more 20-min vigorous activity sessions, five or more 30-min moderate activity sessions, or five or more sessions of any combination of moderate and vigorous sessions. If they do not meet these criteria, they are classified as *insufficiently active*.

We performed a bivariate analysis to investigate associations between independent variables and being in the cancer survivor group or the non-cancer group; the χ^2^ test was used for categorical variables and the *t* test for continuous variables. Statistical significance was established as P < 0.05. Odds ratios with 95% confidence intervals (CIs) were calculated to assess strength of association. Finally, we performed multiple logistic regression analysis of variables with a P value < 0.20 in the bivariate analysis using backward elimination. Whenever a variable was eliminated from the model, changes to the B coefficients were checked to assess confounding. Statistical analyses were performed in SPSS, version 23. The study was approved by the ethics committees at each of the participating centers.

### Results

In total, 4259 people were included in the study: 190 cancer survivors (4.46%; 95% CI 3.82–5.09) and 4069 non-cancer patients. The median time since diagnosis was 6.5 years (interquartile range, 3–10 years).

The sociodemographic variables are summarized in Table 1. Patients who had survived cancer were on average older than those without a history of cancer, but no significant differences were observed for sex or civil status. In the cancer survival group, there were higher proportions of patients not working, patients with long-term sick leave, and patients with primary education or no schooling.

**Table 1:** Sociodemographic characteristics of study participants

Sociodemographic variables	Cancer survivors	Non-cancer patients	P
	N = 190	N = 4069	
	N (%)	N (%)	
Mean (SD) age, years	62.8 (7.5)	58.7 (8.1)	< 0.001
Sex			
Male	85 (44.7)	1.762 (43.3)	0.70
Female	105 (55.3)	2.307 (56.7)	
Civil status			
Single	4 (2.1)	114 (2.9)	< 0.001
Married/living with a partner	61 (32.1)	1.058 (26.8)	
Separated/divorced	102 (53.7)	2.104 (53.3)	
Widowed	16 (8.4)	478 (12.1)	
Employment status			
Not working	119 (72.6)	1.616 (40.9)	< 0.001
Working	51 (26.8)	1.777 (45.0)	
Unemployed	7 (3.7)	413 (10.5)	
Long-term sick leave	13 (6.8)	14 (13.6)	
Level of education			
Higher/university education	28 (14.7)	583 (14.8)	0.004
Secondary education	54 (28.4)	1.523 (38.6)	
Primary education	85 (44.7)	1.573 (39.8)	
No schooling	23 (12.1)	270 (6.8)	

*SD* standard deviation

The results for lifestyle habits, comorbidities, and social functioning are shown in Table 2. Almost four of every 10 patients in the overall group smoked, and while the rate was somewhat lower among cancer survivors, the difference with non-cancer patients was not significant. Adherence to the Mediterranean diet and physical activity levels were also low overall. Almost 80% of the members of both groups had low adherence to the Mediterranean diet, and almost 90% were insufficiently active. Engagement in healthy behaviors was not more common among patients who had survived cancer.

**Table 2:** Distribution of lifestyle behaviors, comorbidities, and social function

Variables	Cancer survivors	Non-cancer patients	OR (95% CI)	P
	N (%)	N (%)		
Lifestyle				
Smoking				
No	120 (63.2)	2.428 (59.7)	1	
Yes	70 (36.8)	1.641 (40.3)	0.86 (0.63–1.16)	0.33
Adherence to Mediterranean diet				
Good (score ≥ 9)	35 (18.4)	646 (16.3)	1	
Low (score 0–8)	155 (81.6)	3.325 (83.7)	0.86 (0.59–1.25)	0.43
Physical activity				
Insufficiently active	168 (89.4)	3.590 (88.8)	1	
Sufficiently active	20 (10.6)	453 (11.2)	1.06 (0.66–1.70)	0.81
Health status and social function				
Self-perceived health				
Excellent/very good/good	42 (44.2)	1.187 (59.1)	1	
Fair/poor	53 (55.8)	823 (40.9)	1.82 (1.02–2.75)	0.005
Weight				
Normal	34 (17.9)	750 (19.0)	1	
Overweight	62 (32.6)	1.442 (36.6)	0.94 (0.61–1.45)	0.80
Obese	94 (49.5)	1.753 (44.4)	1.18 (0.79–1.76)	0.41
No. of health problems				
0	67 (35.3)	1741 (42.8)	1	
1	58 (30.5)	1.325 (32.6)	1.13 (0.79–1.62)	0.48
≥ 2	65 (34.2)	1.003 (24.6)	1.68 (1.18–2.39)	0.004
Depression				
No	166 (87.4)	3.742 (92.0)	1	
Yes	24 (12.6)	327 (8.0)	1.65 (1.06–2.57)	0.02
Hypertension				
No	107 (56.3)	2.506 (61.6)	1	
Yes	83 (43.7)	1.563 (38.4)	1.24 (0.92–1.66)	0.14
Chronic obstructive pulmonary disease				
No	175 (92.1)	3.915 (96.2)	1	
Yes	15 (7.9)	154 (3.8)	2.17 (1.25–3.78)	0.006
Diabetes mellitus not affecting target organs				
No	149 (78.4)	3.405 (83.7)	1	
Yes	41 (21.6)	664 (16.3)	1.41 (0.98–2.01)	0.057
Self-perceived social support				
Standard (score ≥ 32)	180 (95.2)	3.609 (95.4)	1	
Low (score < 32)	9 (4.8)	296 (7.6)	0.61 (0.30–1.20)	0.15

*OR* odds ratio, *CI* confidence interval

Cancer survivors had significantly worse self-perceived health, more comorbidities, and higher rates of depression, diabetes, and chronic obstructive pulmonary disease (COPD). Both groups had similar perceptions of social support. Multivariate analysis confirmed that cancer survivors had worse self-perceived health than patients who had never had cancer (Table 3).

**Table 3:** Multiple logistic regression analysis

Variables	Adjusted OR (95% CI)	P
Age	1.04 (1.001–1.081)	0.014
Self-perceived health		
Excellent/very good/good	1	
Fair/poor	1.81 (1.18–2.78)	0.006
Employment status		
Not working	1	
Working	0.56 (0.29–1.06)	0.07
Unemployment	0.43 (0.15–1.16)	0.09
Long-term sick leave	1.49 (0.67–3.32)	0.32

*OR* odds ratio, *CI* confidence interval

### Discussion

In this study cancer survivors were on average older than non-cancer patients, perhaps because two-thirds of cancer cases occur in patients aged over 60 years. Older age would also explain why cancer survivors were more likely not to be working and to be on long-term sick leave, although the unemployment rate was lower in this group than in the non-cancer group. Other authors have found that cancer survivors experience difficulties returning to their normal lives, and to work in particular [24]. Islam et al. [25] reported that patients with cancer were 1.4 times more likely to be unemployed than healthy patients due to disease- and treatment-related problems, highlighting the importance of support programs to help them return to work when they are able to [26].

Our study shows similar behaviors among cancer survivors and non-cancer patients in terms of exercise, diet, and smoking, with very high levels of unhealthy behaviors and overweight/obesity in both groups. This observation supports previous reports that modifiable cardiovascular risk factors such as hypertension, obesity, smoking, and physical inactivity are more common in cancer survivors than in the general population [27]. One would expect people who have survived cancer to be more motivated to lead a healthy lifestyle and one would also expert them to receive guidance from their health care providers on how to make suitable changes, particularly in terms of diet and exercise, to prevent recurrent and new cancers, reduce cardiovascular risk factors, and improve quality of life [12, 28]. Nevertheless, healthy lifestyle choices among cancer survivors and support from health care providers in making these choices would appear to be suboptimal. Weaver et al. [27] found that just one in three cancer survivors reported having had a health promotion discussion with their health care provider. It has also been found that some survivors are unsure about how to make changes and perceive a lack of support from their health care team [29]. Lifestyle interventions are known to bring about positive lifestyle changes that result in improved health and functioning [30], and these changes should be a primary goal for cancer survivors.

Cancer survivors had worse self-perceived health than non-cancer patients. Depression, COPD, and chronic non-malignant conditions were all more common in this group, supporting previous findings showing a higher prevalence of chronic lifestyle- or treatment-related diseases in cancer survivors compared with members of the general population [6, 30]. No significant differences were observed for obesity, but this may be because both groups had similar obesity-related risk factors.

The cancer survivors perceived a similar level of social support to non-cancer patients. For some authors, social functioning may be affected in the early years of diagnosis and beyond [31–34] but others have found no differences in perceptions of family support or satisfaction with partners between cancer survivors and non-cancer patients [35]. It may be that adults who survive a cancer become more resilient to adverse situations and learn mechanisms to maintain their self-esteem and continue to enjoy good social and personal relationships.

The results of this study indicate a need to encourage cancer survivors to make positive lifestyle changes that will bring them better general health and protect against recurrent and new cancers and other chronic diseases. Primary care providers should systematically evaluate lifestyle behaviors of patients with cancer, advise them on associated health risks and benefits, and encourage them to cultivate healthy habits. Further research is needed to investigate why more cancer survivors do not engage in healthy lifestyle behaviors.

### Conclusions

In this series, we observed that cancer survivors had more chronic diseases and were more likely to be on long-term sick and have worse self-perceived health than people with similar characteristics without a history of cancer. The two groups, however, had similar perceptions of social support. A large proportion of cancer survivors engaged in unhealthy lifestyle practices that could negatively affect both their health and quality of life. Health care providers must take an active role in assessing their patients’ lifestyle habits after a diagnosis of cancer and discuss the beneficial effects that leading a healthy lifestyle can have on quality of life and prognosis.

## Limitations

- The clinical trial participants had to have at least two modifiable risk behaviors and this means that unhealthy behaviors will have been overrepresented in our sample, preventing us from making population-based estimates of differences between cancer survivors and non-cancer patients.
- Worse health status and higher permanent disability rates among cancer survivors could have been influenced by their older age, although it should be noted that poor self-perceived health remained a significant predictor of cancer survivorship after adjusting for confounders.
- We were unable to gain a broader perspective of health problems that may affect cancer survivors because our sample was small, on some of the more uncommon diseases in the CCI.

## Supplementary information

**Additional file 1: Figure S1.** Flow chart of subjects. Phase II and III study EIRA.

## Data Availability

The datasets used and analyzed during the current study are available from the corresponding author on reasonable request.

## Abbreviations

RedIAPP: Primary Care Prevention and Health Promotion Network
GPs: General Practitioners
CCI: Charlson Comorbidity Index
COPD: Chronic obstructive pulmonary disease
SD: Standard deviation
OR: Odds ratio
