# Supplementary material for: Health status, lifestyle habits, and perceived social support in long-term cancer survivors: a cross-sectional study
Source: BMC Res Notes. 2020 Aug 8;13:376. doi: 10.1186/s13104-020-05218-8 (PMC7414741; doi:10.1186/s13104-020-05218-8)
Supplement: Supplementary file 1 — Additional file 1: Figure S1. Flow chart of subjects. Phase II and III study EIRA. [file 13104_2020_5218_MOESM1_ESM.pptx]

## Slide 1
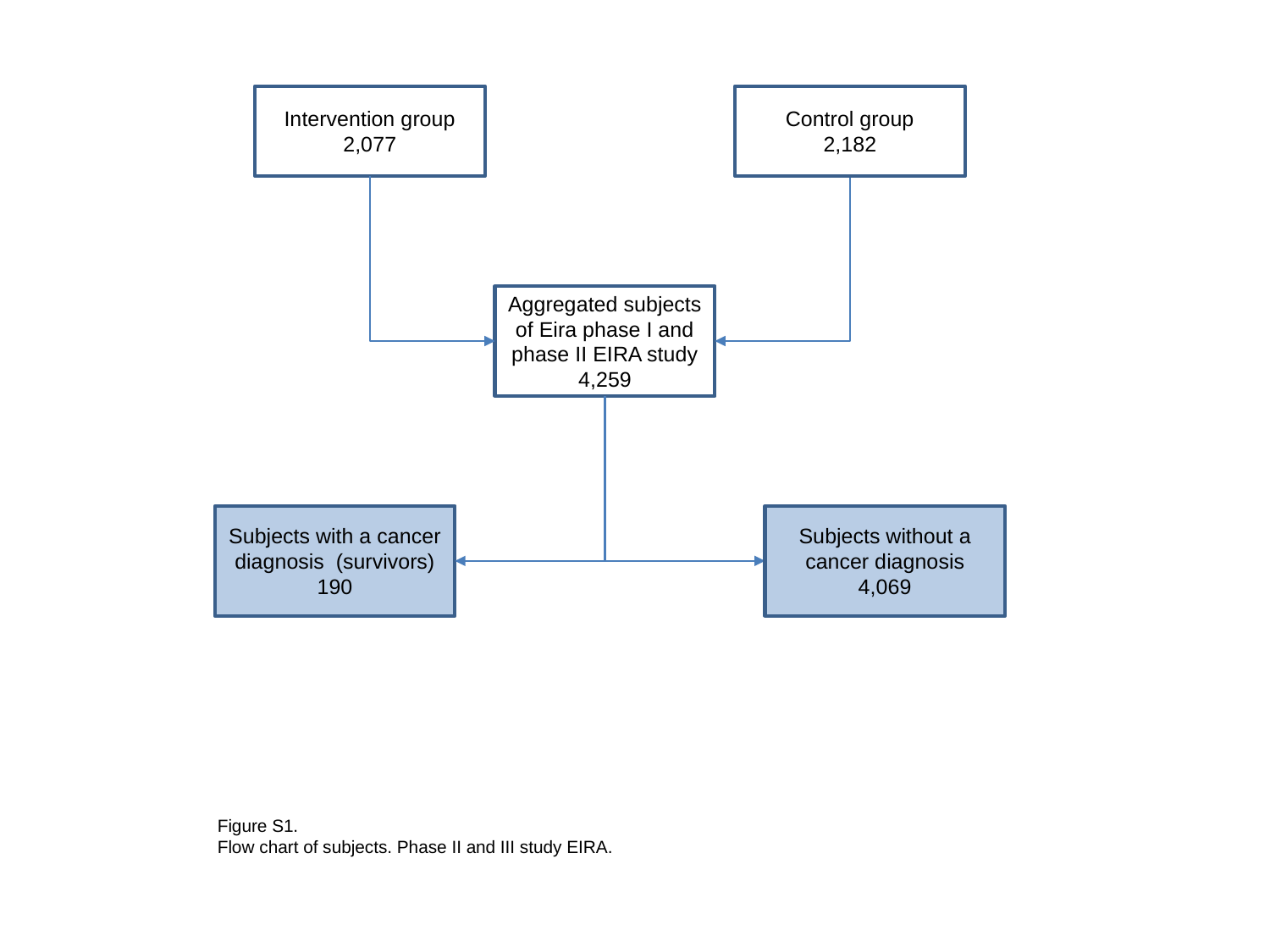

Intervention group
2,077
Control group
2,182
Aggregated subjects of Eira phase I and phase II EIRA study
4,259
Subjects with a cancer diagnosis (survivors)
190
Subjects without a cancer diagnosis 4,069
Figure S1.
Flow chart of subjects. Phase II and III study EIRA.
